# Treatment of pregnant and early postpartum women with severe and critical COVID-19: experience at a tertiary center

**DOI:** 10.1186/s40001-022-00907-5

**Published:** 2022-12-02

**Authors:** Rodrigo Nacif Barbosa, Maria Aparecida Braga, Bárbara Braga Costa, Frederico José Amedee Peret

**Affiliations:** 1 Unidade de Tratamento Intensivo, Maternidade Unimed-Unidade Grajaú, Belo Horizonte, Brazil; 2 Rua Viamão, 1171, UTI adulto. Bairro Grajaú, Belo Horizonte, Minas Gerais CEP 30431-253 Brazil

**Keywords:** Pregnancy, COVID-19, SARS-CoV-2, Acute respiratory failure, Mechanical ventilation, Prone positioning, Delivery

## Abstract

**Background:**

The management of acute respiratory failure during pregnancy is a poorly defined issue in the literature, especially regarding the use of the prone position and the appropriate time for delivery. This study describes our experience in treating pregnant and postpartum women with severe or critical coronavirus disease 2019 (COVID-19).

**Materials and methods:**

This descriptive retrospective study included 25 pregnant and 4 postpartum women admitted to an ICU due to respiratory complications from COVID-19 from June 2020 to August 2021.

**Results:**

The mean maternal age was 33.6 years, and the median gestational age (GA) at admission was 33 weeks. Obesity was the most common comorbidity. The median time between symptom onset and ICU admission was 10 days, while the median length of ICU stay was 14 days. Invasive mechanical ventilation (IMV) was required in 16 (55.2%) patients for a median time of 16.5 days. Prone positioning (PP) was performed in 68.7% of the patients on IMV, and resulted in an expressive increase in arterial oxygen partial pressure to fractional inspired oxygen (PaO2/FiO2 ratio). Eleven (44%) pregnant women delivered during their ICU stay for obstetric or fetal reasons: of these, 2 (18%) developed postpartum hemorrhagic shock and 1 (9%) developed abdominal wall infection. None of the 25 pregnant women underwent delivery due to acute respiratory failure or in an attempt to avoid intubation. There were 2 fetal deaths, but no maternal or neonatal deaths.

**Conclusion:**

We observed favorable outcomes in pregnant and postpartum women with severe and critical COVID-19 admitted to our institution. This finding reinforces the effectiveness of PP in the treatment of hypoxemic respiratory failure secondary to COVID-19 in pregnant women undergoing IMV, and suggests that gestation should only be interrupted in cases of obstetric and fetal complications, provided the patient is stable, or when hypoxemia is refractory to PP.

## Introduction

The COVID-19 pandemic has caused millions of deaths throughout the world; as of this writing (February 6, 2022), 5.75 million deaths have been reported worldwide, 631 000 in Brazil [1]. On the same date, a Brazilian database recorded 20,176 cases of severe acute respiratory syndrome due to COVID-19 with a lethality rate of 8.1% in pregnant women and 17.3% in postpartum women [2]. In addition to this difference between pregnancy and postpartum women, lethality in both these populations nearly doubled in 2021 compared to 2020. This intensification was attributed to the collapsed healthcare system during the second COVID-19 wave in Brazil caused by the Gamma variant and its likely greater virulence. [3, 4]

Cohort studies and case series on intensive care for pregnant women with critical COVID-19 have been published [5–9], raising questions about different ventilatory and obstetric management. Pregnant and postpartum women were excluded or not mentioned in the main studies on IMV in acute respiratory distress syndrome [10–12] as well as several large studies on COVID-19 [13–16]. We are not aware of any reports on pregnant and postpartum women with severe or critical conditions treated in the ICU while the Gamma variant was dominated before this study was conducted.

## Objective

This study describes the clinical characteristics and clinical outcomes of pregnant and postpartum women with COVID-19 who received intensive care and shares our experience treating this population.

## Materials and methods

This descriptive, retrospective, single-center study was conducted in pregnant and postpartum women admitted to an ICU in Belo Horizonte, Brazil, due to COVID-19 from June 2020 to August 2021. Patients with respiratory symptoms requiring monitoring and treatment in the ICU for  > 24 h were included in the study; patients with a length of ICU stay of  ≤ 24 h or who were admitted to the hospital for reasons unrelated to respiratory complications from COVID-19 were excluded.

The data were collected from the hospital’s electronic medical records. Data on comorbidities, GA at ICU admission, drugs utilized (corticosteroids, antibiotics, amines, and heparin), lymphocyte count and C-reactive protein levels at admission, acute renal dysfunction during hospitalization, platelet count  < 100 000/mm^3^ during hospitalization, type of respiratory support used (oxygen therapy by nasal cannula [NC] or non-rebreather [NRB] mask with reservoir bag, high-flow nasal cannula [HFNC], noninvasive mechanical ventilation [NIV], and IMV), IMV days, PP, GA at delivery, maternal and fetal complications, fetal mortality (miscarriage and stillbirth), and neonatal and maternal mortality were obtained.

From June 2020 to August 2021, our ICU received patients from our own hospital as well as from others in the metro region. Most were receiving noninvasive oxygen therapy (NC or NRB) at admission; one was admitted with intense dyspnea at rest but without hypoxemia, and only one was already intubated upon admission.

## Institutional protocol

- Peripheral O_2_ saturation was maintained at 95% or above.
- Self-proning in awake, non-intubated patients was encouraged in all cases with greater need for oxygen therapy or when respiratory distress or effort was perceived as moderate or intense.
- Protective IMV with a tidal volume of 6 mL/kg, plateau pressure (Pplat) of  ≤ 30 cmH_2_O, and driving pressure of  ≤ 15 cmH_2_O was intended in all intubated patients. These limits were exceeded only in the case of respiratory acidosis with pH of  < 7.2 after respiratory rate (RR) optimization. The positive end-expiratory pressure (PEEP) associated with best static compliance of the respiratory system was defined by a decremental method, never preceded by alveolar recruitment.
- PP was performed in all intubated patients who maintained a PaO_2_/FiO_2_ ratio of  < 150 after a stabilization period regardless of GA or fetal conditions. Each PP session usually lasted 18 h.
- Prophylactic enoxaparin was used in all patients.
- Corticosteroids were used in all patients who required supplemental O_2_. Methylprednisolone was administered at a dose of 40 mg every 12 h and was subsequently reduced to 20 mg every 12 h when the ventilatory parameters improved or septic shock or severe ventilation-associated pneumonia (VAP) occurred. Some patients received dexamethasone 6 mg every 24 h. Faced with the possibility of premature delivery before 34 weeks, the patients initially using methylprednisolone received dexamethasone at a dose of 6 mg every 12 h for 2 days for fetal maturation, provided that there was no urgency for delivery.
- Antibiotics were maintained in patients already using them for more than 48 h, as this time of use hindered the assessment of bacterial coinfection. Otherwise, onset was guided by radiological, clinical, and laboratory findings that included procalcitonin level.
- Conservative fluid management strategy was utilized to achieve a near-zero water balance through drug infusion in concentrated solutions and frequent use of furosemide.
- Obstetric status was assessed at least once a day, with periodic ultrasound. Continuous fetal monitoring was not available in our ICU.

### Statistical analyses

Categorical variables were expressed as absolute (*n*) and relative (%) frequencies. Continuous variables with normal distribution based on the results of the Shapiro–Wilk test were expressed as means and standard deviations, while those with non-normal distribution were expressed as medians and interquartile ranges (IQRs).

## Results

Thirty-four patients (30 pregnant and 4 early postpartum) with COVID-19 were admitted to the ICU during the study period. Five pregnant patients were excluded from the analysis: one had suspected hemolysis, elevated liver enzymes, and low platelet count (HELLP) syndrome without pulmonary involvement due to COVID-19; three had an ICU stay of  < 24 h and were discharged; and one developed a respiratory condition due to asthma exacerbation.

Table 1 shows the maternal and obstetric characteristics. The mean maternal age was 33.6 years, with 41.4% of the women presenting advanced maternal age (> 34 years) and 51.7% having comorbidities, with obesity being the most common. The median GA at admission was 33 weeks. Of the 25 pregnant women, 64% had a GA of  < 34 weeks. The 4 postpartum women were in the immediate puerperal period (up to the 10th postpartum day).

**Table 1:** Maternal and obstetric characteristics^a^

Characteristics	*n* = 29
Pregnant women	25 (86.2%)
Postpartum women	4 (13.8%)
Maternal age	33.6 ± 4.5
< 35 years	17 (58.6%)
≥ 35 years	12 (41.4%)
Comorbidities	
Yes	15 (51.7%)
No	14 (48.3%)
Class 1 or 2 obesity	11 (37.9%)
Class 3 obesity	2 (6.9%)
Previous diabetes	2 (6.9%)
Gestational diabetes	3 (10.3%)
Chronic hypertension	2 (6.9%)
Previous thromboembolism	2 (6.9%)
Parity	
Primiparous	13 (44.8%)
Multiparous	16 (55.2%)
GA on admission	33 (29–35)
< 24 weeks	3 (10.3%)
≥ 24 to < 34 weeks	13 (44.8%)
≥ 34 weeks	9 (31.0%)
Postpartum period	4 (13.8%)
Vaccination status	
No dose	28 (96.6%)
One dose (CoronaVac vaccine)	1 (3.4%)

^a^Data presented as mean ± standard deviation or median (IQRs) or *n* (%)

Table 2 shows the clinical characteristics of the population. The median time from symptom onset to ICU admission was 10 days. The median lymphocyte count and C-reactive protein level on admission were 911/mm^3^ and 80 mg/L, respectively. In 3 patients, platelet count dropped below 100,000/mm^3^ during ICU stay: in 2 due to sepsis, and in 1 due to HELLP syndrome. Only 1 of the 29 patients did not require oxygen therapy, but was monitored in the ICU due to significant dyspnea. Of the 18 patients using HFNC or NIV, 12 (66.6%) required intubation. In addition to these patients, 3 were intubated using NRB at 15 L/min and 1 patient was already intubated upon admission, totaling 16 patients; of these, 15 (93.1%) used neuromuscular blocking agents (NMBAs) for a mean of 8.1 days. The median time from symptom onset to intubation was 12 days, and the median time on IMV was 16.5 days. Of the 16 intubated patients, 7 (43.7%) required tracheostomy (TT) after a mean of 16.7 days on IMV, and 11 (68.7%) underwent a total of 41 PP sessions, but the mean duration of the proning sessions was not measured. The median lengths of ICU and hospital stay of the total sample were 14 and 20 days, respectively. Two fetal deaths were reported, but no maternal or neonatal deaths.

**Table 2:** Clinical characteristics and results^a^

Characteristics	*n* = 29
Time from symptom onset to ICU admission; days	10 (8.5–11)
Lymphocyte count on admission; /mm^3^	911 (708–1185)
C-reactive protein level on admission; mg/L	80 (62.0–170.5)
Platelet count < 100 000/mm^3^ during ICU stay	3 (10.3%)
Respiratory support	
None	1 (3.4%)
NC or non-rebreather mask with reservoir bag	28 (96.6%)
HFNC and/or NIV	18 (62.1%)
Subsequent intubation	12 (41.4%)
No subsequent intubation	6 (20.7%)
IMV	16 (55.2%)
Time from symptom onset to intubation; days	12 (10.2–14.0)
IMV time; days	16.5 (9.2–22.0)
PEEP associated with best compliance (cmH_2_O)	11 (10–12)
PP in intubated patients	11 (37.8%)
Number of sessions per patient	2 (1–8)
PaO_2_/FiO_2_ before PP	105.6 ± 22.2
PaO2/FiO2 during first PP session	262.9 ± 77.7
ECMO*	0 (0.0%)
Tracheostomy	7 (24.1%)
Time between TI^†^ and TT^‡^; days	16.7 ± 4.9
Continuous sedation	16 (55.2%)
NMBA	15 (51.7%)
Neuromuscular blocking time; days	8.1 ± 4.6
Amines	
Noradrenaline	12 (41.4%)
Dobutamine/epinephrine/dopamine/phenylephrine	0 (0.0%)
Transfusion; number of patients	4 (13.8%)
Corticosteroids	29 (100%)
Corticosteroid time; days	12 (10–18)
Antibiotics	27 (93.1%)
Anticoagulants	
Prophylactic enoxaparin	27 (93.1%)
Therapeutic enoxaparin	2 (6.9%)
Remdesivir, tocilizumab	0 (0.0%)
Length of ICU stay	14 (4.5–23.0)
Length of hospital stay	20 (11.0–31.5)
Death	
Maternal	0 (0.0%)
Fetal (stillborn)	2 (6.9%)
Neonatal	0 (0.0%)

^a^Data presented as mean ± standard deviation or median (IQRs) or *n* (%)

^*^ECMO, extracorporeal membrane oxygenation

^†^TI, tracheal intubation

^‡^TT, tracheostomy

Table 3 shows obstetric outcomes of the 25 pregnant women. During ICU stay, 11 (44%) women delivered at a mean GA of 35.6 days, with 9 delivering via cesarean section. Delivery was indicated for obstetric or fetal reasons.

**Table 3:** Obstetric outcome of pregnant women^a^

Characteristic	*n* = 25
Delivery during ICU stay	11 (44%)
GA at delivery	35.6 ± 2.8
Vaginal delivery	2 (8%)
Cesarean section	9 (36%)
Delivery at GA of < 37 weeks	5 (20%)
Indications for delivery during ICU stay	
Respiratory failure	0 (0.0%)
Fetal distress	4 (16%)
PROM*	3 (12%)
PROM and fetal distress	1 (4%)
Labor and HELLP syndrome	1 (4%)
Fetal death	2 (8%)

^a^Data presented as mean ± standard deviation or *n* (%)

^*^PROM, premature rupture of membranes

Table 4 presents the data on maternal complications. The most frequent were VAP, acute renal failure, and urinary tract infection. Two patients developed thromboembolic complications. Two (6.9%) patients experienced postpartum hemorrhagic shock, while one (3.4%) developed abdominal wall infection, corresponding to 18% and 9% of patients who underwent delivery during ICU stay, respectively.

**Table 4:** Maternal complications^a^

Maternal complications	*n* = 29
VAP	11 (37.9%)
VAP causative pathogens	
XDR^*^ *Acinetobacter baumannii*	1 (3.4%)
MDR^†^ *Acinetobacter baumannii*	4 (13.8%)
MS^‡^ *Acinetobacter baumannii*	1 (3.4%)
MS *Staphylococcus aureus*	2 (6.9%)
* Serratia marcescens*	1 (3.4%)
MS *Klebsiella pneumoniae*	1 (3.4%)
MS *Enterobacter cloacae*	1 (3.4%)
Acute renal failure (KDIGO)^§^	10 (34%)
Stage 1	4 (13.8%)
Stage 2	5 (17.2%)
Stage 3	1 (3.4%)
Renal replacement therapy	0 (0.0%)
Catheter-related bloodstream infection	1 (3.4%)
Catheter-associated urinary tract infection	6 (20.7%)
Pressure injury stage 2 or higher	4 (13.8%)
Pulmonary embolism	1 (3.4%)
Internal jugular and subclavian vein thrombosis	1 (3.4%)
Acute biliary pancreatitis	1 (3.4%)
COVID-19 related acute pancreatitis	1 (3.4%)
Upper limb bacterial cellulitis	2 (6.9%)
Abdominal wall infection after cesarean section	1 (3.4%)
Partial intestinal obstruction	1 (3.4%)
Postpartum hemorrhagic shock	2 (6.9%)

^a^Data presented as n (%)

^*^XDR, extensively drug resistant

^†^MDR, multidrug resistant

^‡^MS, multidrug susceptible [17]

^§^Kidney disease: improving global outcomes [18]

## Discussion

Of the 29 patients included in the study (25 pregnant and 4 postpartum women), 25 (86%) were admitted from March 2021 to August 2021, when the ICU was used exclusively for the treatment of COVID-19. During this period, the Gamma variant became predominant in Belo Horizonte [19], which was detected in 82%, 93%, and 100% of genotyping results in March, April, and May 2021, respectively.

Most of the patients were admitted to the ICU already receiving antibiotics despite reports of infrequent bacterial coinfection [20–22]. Antibiotics were maintained in patients who had been using them for more than 48 h, since prolonged use could interfere in the evaluation of possible coinfection and increase the risk of false negatives. In addition, a procalcitonin level of  < 0.25 ng/ml, which has shown a high negative predictive value for bacterial coinfection [23–25], could reflect a regressing bacterial infection state, and early discontinuation of antibiotics could worsen the patient’s condition.

Remdesivir or tocilizumab was not used due to the lack of availability. Corticosteroids were administered to all patients, although one patient did not require supplemental O_2_. In most patients (76%), the corticosteroid dose was higher than that used in the RECOVERY TRIAL [26] in view of the assumption that higher doses could provide better results in a disease with such an inflammatory response. However, up to the time of this study this issue remains unclarified [13, 27, 28].

Failure of the instituted respiratory support was defined by the attending physician using objective and subjective criteria and always considering the oxygenation level, RR, and respiratory effort. The ROX index, defined as SpO2/FiO2/RR, was not used due to the lack of reports in pregnant women [29]. All 16 patients on IMV were intubated for respiratory failure, and there was no intubation due to shock or organ failure.

As mentioned, reports of ventilatory support in pregnant women with hypoxemic respiratory failure and acute respiratory distress syndrome (ARDS) are scarce. Protective IMV has become the state-of-the-art for acute respiratory failure in general, and its use in patients with respiratory failure due to COVID-19 has indicated lower mortality [30].

The best method for determining the optimal PEEP has not been established in the literature and remains under debate. We chose to determine it through the PEEP associated with the best static compliance of the respiratory system by the physiological appeal of the method, which appears to show a better relationship between pulmonary overdistension and collapse, allowing the highest tidal volume with the lowest driving pressure. Using this method, the median PEEP in our patients was 11; however, comparison with other studies, even in pregnant women, cannot be performed because the PEEP value is established by the method used for its determination. For example, a study that excluded pregnant women used a median PEEP of 15 cmH2O (IQR: 13.5–16) obtained with a higher-PEEP strategy [31]. Even considering the need to increase FiO_2_, we maintained PEEP at the value corresponding to the best compliance, and prioritized PP to improve oxygenation when the PaO2/FiO2 ratio fell below 150.

PP is now an integral part of protective IMV and has been shown to improve oxygenation in patients with COVID-19 [15, 31, 32] and to probably reduce mortality [15]. Again, these studies excluded or did not mention pregnant women. Although a previous study [33] has demonstrated that PP in healthy pregnant women improves umbilical artery flow by the decompressive effect of the uterus on the large abdominal vessels, this important technique in the treatment of acute respiratory failure has been cautiously used in this group of patients. A recent study has demonstrated favorable maternal and fetal hemodynamic effects during PP [34]. In terms of oxygenation improvement, the potential effect of PP on pregnant women may be greater than in non-pregnant women, since the pulmonary compression by the diaphragm, caused by the greater content and greater abdominal pressure in pregnant women, is relieved by PP, especially in its posterior portion, where the largest amount of pulmonary collapse occurs. The first case reports on PP as a treatment method in pregnant women with ARDS were published in 2009 [35] and 2014 [36]. Several reports and studies demonstrated the use of PP with reservations. Some researchers subjected patients to extracorporeal membrane oxygenation (ECMO) without previous PP attempts [37–39] and others performed cesarean section before PP [40]. In a study on ECMO in pregnant and puerperal women with ARDS, PP was attempted before ECMO in only 2 of the 7 pregnant women with respiratory failure [38]. A more recent publication showed a greater tendency to use PP before ECMO in pregnant women with COVID-19; nevertheless, it was not attempted in 42% of the 100 pregnant and postpartum women in the study [39].

Case reports and series have shown the successful use of PP in pregnant women with COVID-19 [6, 7, 41, 42]. In our series, of the 16 intubated patients, 11 (69%) required PP according to the criterion used, which is similar to that described by Guérin et al. [10], except for the stabilization time in MV before its indication, which sometimes was  < 12 h. The median of PP sessions per patient was two. Four patients (36% of patients in PP) required only one PP session. At the other end of severity, 2 patients required 48 continuous hours of PP due to severe and immediate refractory hypoxemia when placed in supine position. Although all 11 patients responded to PP (8 pregnant and 3 postpartum women), data on the PaO_2_/FiO_2_ ratio were obtained before and during PP in only 10 of them (7 pregnant and 3 postpartum women). Two patients had a PaO_2_/FiO_2_ ratio between 80 and 100 before PP, and 2 others had a PaO_2_/FiO_2_ ratio of  < 80. All patients responded with an expressive increase in this ratio; however, the magnitude of this increase may have been influenced by the time of PaO_2_/FiO_2_ measurement when in PP, which ranged from 2 to 12 h. The lowest response was a 38% increase, in a 29 week pregnant woman. The only maternal complications observed were facial edema and superficial skin lesions.

Our report reinforces the effectiveness of PP in the treatment of hypoxemic respiratory failure in pregnant women undergoing IMV. A recent study reported that the procedure was safe in a series of 17 pregnant women under IMV for COVID-19, 13 of whom were proned under continuous fetal monitoring and tocodynamometry, with no fetal intolerance observed [9]. However, these authors did not find a positive effect on oxygenation because they used PP as a preventive measure.

Also related to protective IMV and Pplat, some authors have suggested tolerating pressure of up to 35 cmH_2_O [43], although this is not a consensual recommendation [44]. In our series, a Pplat of  ≤ 30 cmH_2_O and a driving pressure of  ≤ 15 cmH_2_O were maintained in 15 of the 16 patients on IMV, controlling respiratory acidosis by increasing the minute volume through the elevation of the RR up to 35 breaths per minute, taking care to not increase total PEEP. The only patient who required a Pplat of  > 30 cmH_2_O was a pregnant woman who had a static lung compliance of 16 cmH_2_O and severe respiratory acidosis with a pH level of  < 7.2 despite an RR of 35 breaths per minute. She required 14 PP sessions, 30 days of IMV, and delivered due to fetal distress identified 2 days after finishing the PP sessions. The fetus progressed well, and the patient experienced hemorrhagic shock due to late intra-abdominal bleeding and was submitted to reoperation for uterine suture.

The IMV time in our series was longer than that in other reports [8, 9], perhaps due to the clinical differences inherent to the prevalent variant; however, there is a lack of data to support this hypothesis. Another reason could be the prolonged use of NMBAs in our intubated patients. Additionally, an outbreak of multidrug resistant (MDR) *Acinetobacter baumannii* occurred in our unit from April to May 2021, which caused VAP in 5 of our patients; this may partly explain our longer time on IMV [45], particularly during a shortage of polymyxin caused by the high demand during the pandemic, which delayed treatment of 4 patients with VAP caused by MDR Acinetobacter who had to be treated with an alternative antimicrobial regimen until polymyxin was available. Mean IMV times in patients with and without VAP due to MDR *Acinetobacter baumannii* were 28.8 days and 12.2 days, respectively.

One of the most challenging issues regarding critical care in pregnant women is the appropriate time for delivery. Although delivery can improve the respiratory condition of some patients with respiratory failure, not all demonstrate improved respiratory mechanics [46], especially when the respiratory failure is not caused by an obstetric complication [47], as is the case with COVID-19. Furthermore, rapid postpartum respiratory worsening is frequently reported in COVID-19 patients [37, 48–51]. The possible explanations for this worsening include increased plasma volume, decreased colloid osmotic pressure, and increases in inflammatory factors such as interleukin-6, which occur during and after delivery [52]. Interestingly, we noted that the respiratory status of 2 of the 4 early postpartum patients in our series worsened acutely during the first hours after delivery, while the remaining 2 developed respiratory failure 1 day after the onset of symptoms. All 4 were intubated and 3 required PP. None had previous comorbidities, and 3 were aged  < 35 years. Despite this finding, it was not possible to confirm if the clinical worsening was due to changes in maternal condition or to the natural course of the disease.

In addition to the risk of respiratory worsening, reports and studies based on large databases showed significantly higher postpartum case-fatality rate from COVID-19 in Brazil [53–55]. Some hypotheses for this poorer prognosis include more frequent cesarean delivery, cardiorespiratory and inflammatory changes triggered in the postpartum period, potential complications from delivery at a time of greater disease severity, and the longer delay in seeking medical assistance. Although COVID-19 increases the risk of fetal distress and death [56, 57], it also significantly increases the risk of postpartum bleeding [56], which has been reported in up to 29% of critically ill patients on IMV [9]. Furthermore, the risk of postpartum infections such as endometritis and abdominal and pelvic infection should also be considered. These issues, associated with the reports of acute exacerbation of the respiratory condition in the postpartum period and lack of an adequate method to predict which patient will present respiratory improvement after delivery, must be considered when deciding whether delivery is indicated in patients with severe or critical COVID-19. Therefore, we believe that delivery should be delayed in intubated or non-intubated patients who are in a worsening phase of COVID-19 or who are receiving intense respiratory and hemodynamic support, even if they appear to be “stable”.

Unlike other studies [5, 8], it was not necessary to indicate delivery for maternal reasons such as respiratory worsening in our series. We credit this result to the frequent use of PP and its excellent response in oxygenation. We observed that PP was a lifesaving method for some patients and believe it should be instituted regardless of GA and following the same recommendations as in non-pregnant women, since it is practically costless and effective with low risk of complications when performed by a properly trained team. It is also likely that PP reduces the need for patients to undergo ECMO, with its potentially severe complications, as long as ECMO is not indicated for hemodynamic issues.

All our indications for delivery were made for fetal or obstetric reasons. Furthermore, during fetal distress, delivery was only performed if the maternal clinical condition had been satisfactorily stabilized with a low noradrenaline dose and adequate ventilatory support, so that we could take action if the mother's condition did worsen.

Our series reported two fetal deaths. One occurred at a 38-week gestation when the patient went into septic shock. The fetus was extracted vaginally, the patient's ventilatory status did not improve after delivery, and PP was still required for the subsequent 4 days. The other occurred at a 37-week gestation and was identified during the second PP session; the fetus was extracted through cesarean section after 11 days, when the patient had already been extubated. The moment of severe fetal bradycardia was identified hours prior, but the mother's condition at that time did not allow an emergency cesarean, because when placed in the supine position, her pH was below 7.20 and FiO_2_ need was 80%. For this reason, there is no way to determine whether PP was related to this event, or whether the fetal death was caused by the viral infection or even the maternal hemodynamic instability.

This study has some limitations. Although epidemiological data show the Gamma variant predominated in our city from March 2021, genotyping was not conducted in our patients. We did not follow patients and live births over the long term. Because we did not utilize continuous fetal monitoring, our observations about fetal repercussion in critical COVID-19 are limited. Chest computed tomography angiography was rarely used in patients with worsening respiratory condition, which may have underestimated the diagnosis of pulmonary embolism.

## Conclusion

We observed favorable outcome in pregnant and early postpartum women with severe and critical COVID-19 in our ICU, both before and during the spread of the Gamma variant in our city. Our findings reinforce the effectiveness of PP in the treatment of hypoxemic respiratory failure secondary to COVID-19 in pregnant and postpartum women undergoing IMV. In our experience, pregnancy should only be interrupted when obstetric or fetal complications develop, provided the mother is stable, or in cases where hypoxemia is refractory to PP. However, more studies are needed to better determine the appropriate timing of delivery in pregnant women with COVID-19 related hypoxemic respiratory failure and the possible fetal repercussions of maintaining a pregnancy during critical COVID-19.

## Data Availability

According to institution regulation, information concerning each patient’s results cannot be published. Nevertheless, the data that support the findings of this study are available upon request at Dr. Rodrigo Nacif Barbosa at rodrigonacif@gmail.com.
